# The costs of *Suaahara* II, a complex scaled‐up multisectoral nutrition programme in Nepal

**DOI:** 10.1111/mcn.13658

**Published:** 2024-05-05

**Authors:** Esther M. Choo, Christopher G. Kemp, K. C. Sagun, Uttam Paudel, Jolene Wun, Kenda Cunningham, Pushpa Acharya, Pooja Pandey Rana, Carol Levin

**Affiliations:** ^1^ Department of Global Health University of Washington Seattle Washington USA; ^2^ Department of International Health Johns Hopkins University Baltimore Maryland USA; ^3^ Helen Keller International Patan Nepal; ^4^ Independent consultant Patan Nepal; ^5^ Independent Consultant Washington District of Columbia USA

**Keywords:** costing, economic evaluation, multisectoral nutrition, Nepal, time use

## Abstract

Limited evidence exists on the costs of scaled‐up multisectoral nutrition programmes. Such evidence is crucial to assess intervention value and affordability. Evidence is also lacking on the opportunity costs of implementers and participants engaging in community‐level interventions. We help to fill this gap by estimating the full financial and economic costs of the United States Agency for International Development‐funded *Suaahara* II (SII) programme, a scaled‐up multisectoral nutrition programme in Nepal (2016–2023). We applied a standardized mixed methods costing approach to estimate total and unit costs over a 3.7‐year implementation period. Financial expenditure data from national and subnational levels were combined with economic cost estimates assessed using in‐depth interviews and focus group discussions with staff, volunteers, community members, and government partners in four representative districts. The average annual total cost was US$908,948 per district, with economic costs accounting for 47% of the costs. The annual unit cost was US$132 per programme participant (mother in the 1000‐day period between conception and a child's second birthday) reached. Annual costs ranged from US$152 (mountains) to US$118 (plains) per programme participant. Personnel (63%) were the largest input cost driver, followed by supplies (11%). Community events (29%) and household counselling visits (17%) were the largest activity cost drivers. Volunteer cadres contributed significant time to the programme, with female community health volunteers spending a substantial amount of time (27 h per month) on SII activities. Multisectoral nutrition programmes can be costly, especially when taking into consideration volunteer and participant opportunity costs. This study provides much‐needed evidence of the costs of scaled‐up multisectoral nutrition programmes for future comparison against benefits.

## Key messages

- The average unit cost of *Suaahara* II, a scaled up multisectoral nutrition programme covering 42 of 77 districts in Nepal, was US$132 per programme participant (mother in the 1000‐day period) and US$76 per mother–child pair reached per year.
- Community events designed to improve access to and demand for nutritious foods accounted for the largest share of activity costs (29%).
- Opportunity costs of volunteer and participant time are high, accounting for 47% of total costs.
- Female Community Health Workers, already engaged in community nutrition activities, spent significant time on implementing *Suaahara* II activities at 27 h per month.
- Complex multisectoral programmes often require significant resources to ensure coordination and sufficient technical support at national and subnational levels.

## Graphical abstract

*Suaahara* II, a scaled up multisectoral nutrition programme in Nepal, cost US$132 per programme participant (mother in the 1000‐day period). Opportunity costs of volunteer and participant time are high, accounting for 47% of total costs. Complex multisectoral programmes require significant resources to ensure coordination and support at national and subnational levels.

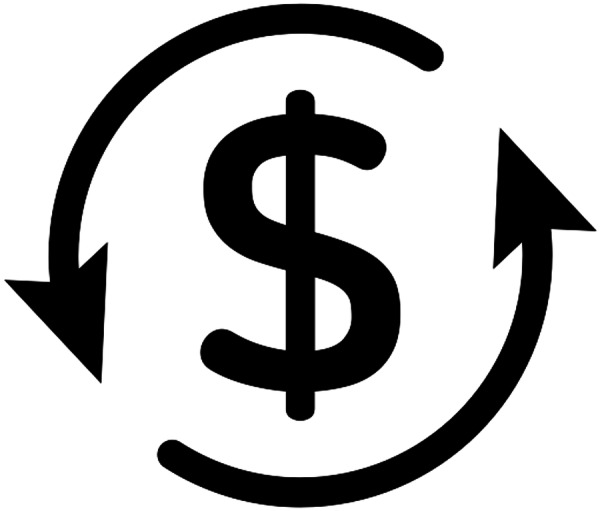

## 1 INTRODUCTION

Nepal has made tremendous progress over the last few decades on many maternal and child health and nutrition indicators (Conway et al., 2020). Commitment to nutrition has been demonstrated by the government's early engagement in the Scaling Up Nutrition global community and its adoption of a Multisector Nutrition Plan (MSNP I 2013‐2017 and MSNP II 2018‐2022) (Government of Nepal, 2012, 2017). The MSNP includes both nutrition‐specific and nutrition‐sensitive strategies, and is currently being implemented throughout the country, including at subnational levels. Since the early 1980s, Nepal has relied on a cadre of more than 52,000 female community health volunteers (FCHVs) to reach communities and households, as much of the population is geographically disconnected from health facilities (New ERA & USAID, 2008). FCHVs have played an important role in delivering community‐based health services and achieving nutrition targets in Nepal. Recent donor investments have leveraged the existing FCHV network and government health programmes to deliver quality health and nutrition programmes.

*Suaahara* II (2016–2023) (SII), a United States Agency for International Development (USAID)‐funded programme, was led by Helen Keller International (HKI), along with six other national and international nongovernmental organizations (iNGO) and 40 Nepali district‐level NGO partners. SII supported Nepal's MSNP, using FCHVs as a central delivery strategy, and built off of gains from *Suaahara* I (2011–2016) and existing government programmes (USAID Nepal, 2017). SII's expanded multisectoral nutrition strategies included interventions in agriculture, water, sanitation, and hygiene (WASH), health, nutrition, gender, and governance in 42 of Nepal's 77 districts, with an aim to reduce maternal and child undernutrition (Supporting Information S1: Appendix Figure 1).

Programme components included multisectoral social behaviour change communication (SBCC), as well as interventions to strengthen facility and community level health and nutrition services; facilities and environments for WASH; and governance at local, district and national level. Targeting households in the 1000‐day period (conception until a child's second birthday), SII explicitly targeted disadvantaged households with an enhanced set of interventions including the addition of homestead food production (Supporting Information S1: Appendix Figure 2). A detailed list of government partners, NGO staff, frontline workers, including community volunteers and their roles in implementing this project can be found in Supporting Information S1: Appendix Table 1. Frontline workers included government volunteers such as FCHVs and SII volunteers such as Village Model Farmers (VMFs) involved in community‐level agriculture activities.

The large scope of this programme—both in terms of sectors and geographic breadth—provides a unique opportunity for global learning about costs of a scaled‐up multisectoral nutrition programme (Ogada et al., 2021). Multisectoral nutrition programmes have complex designs involving multiple partners and activities across sectors, making the comparison of costs challenging (Heckert et al., 2020; Wun et al., 2022). To make the case for further investment in multisectoral nutrition programmes, greater understanding of their costs is needed (Ruel & Alderman, 2013; Ruel et al., 2018). There are significant gaps in knowledge around the true costs of integrated approaches to improve nutrition (Cobiac et al., 2013; Gillespie & van den Bold, 2017; Hawkes et al., 2012; Menon et al., 2014). Earlier systematic reviews of costs of programmes indicated that most studies were modelled and not based on empirical data, or they were conducted in a specific context, not at scale (Fiedler & Puett, 2015; Fiedler et al., 2008; Gyles et al., 2012). More recent reviews indicate that research is still focused on a single nutrition specific intervention and not multisectoral approaches at scale (Baek et al., 2022; Njuguna et al., 2020; Ramponi et al., 2021). All reviews have noted methodological variations that limit the ability to make direct comparisons of costing studies and hinder planning and designing national programmes. Thus, a key challenge in much of the published literature to date has been the lack of comparability, transparency, and transferability. Two recent papers have applied a standard approach to estimating costs of multisectoral nutrition approaches, however these were for smaller projects, not at scale (Margolies et al., 2021; Thai et al., 2023). Although evidence on costs of complex multisectoral programmes at scale remains limited, donors are increasingly requiring or recommending implementers to track and report costs (Ruel & Alderman, 2013; Sharp et al., 2019; USAID, 2014). An understanding of the major cost drivers of SII—by input, activity, and sector—could inform resource allocation for design and implementation decisions and provide budget estimates across relevant sectors for future programmes.

Complex nutrition programmes like SII require a mix of implementing partners, including volunteers, and rely on the engagement of targeted participants and their communities to improve nutrition outcomes. Capturing economic costs (opportunity cost of time and out of pocket expenses), therefore, is essential for assessing full resource use, providing insights into participants’ tradeoffs that can affect programme success. This analysis estimates the time required by government workers, volunteers, programme participants, and NGO staff, providing important insights to programme designers and implementers of multisectoral programmes.

This paper measures the costs of SII, a multisectoral programme in Nepal that reached over two million direct participants including: (1) estimating the total incremental costs and average cost per output and (2) estimating opportunity costs of individual programme participants.

## 2 METHODS

### 2.1 Overall approach

We applied the Strengthening Economic Evaluations in Multisectoral Strategies for Nutrition (SEEMS‐Nutrition) standardized costing approach to understand the costs of SII (Margolies et al., 2021; Thai et al., 2023). The SEEMS‐Nutrition approach was built on and incorporates best practices from the Global Health Cost Consortium Reference Case to estimate SII total and unit costs (Vassall et al., 2017). Total SII costs were measured over and above the investments made in *Suaahara* I; we therefore describe the cost estimates as total incremental (Levin, Masters, et al., 2019).

We followed four stages of the SEEMS‐Nutrition approach. In the first stage, we aligned SII intervention components to a standardized multisectoral intervention typology inspired by the SUN Compendium of Actions for Nutrition and the nutrition‐sensitive value chain (NSV) classification: (1) increasing supply of nutritious foods, (2) increasing demand for nutritious foods and (3) strengthening the enabling environment for nutrition (de la Pena et al., 2018; UN Network for SUN/REACH Secretariat, 2016). The second stage used the programme impact pathway to align programme activities and inputs with the NSV typology (Supporting Information S1: Appendix Figure 1). The third stage estimated costs by programme impact pathway to achieve outputs and outcomes. The last stage combined estimates of total incremental costs with programme outputs to derive unit costs.

Costs were categorized as standardized SEEMS‐Nutrition inputs, activities, NSV typology and sector categories, and as either start‐up or recurrent. Table 1 shows SII activities mapped to the SEEMS‐Nutrition cost categories by activity and NSV typology. Start‐up costs included programme design, initial trainings, materials development, and programme sensitization. Recurrent costs included home visits, community groups and events, field supervision, management and refresher trainings. Supporting Information S1: Appendix Table 2 describes standard input cost categories.

**Table 1:** SII activities mapped to SEEMS‐Nutrition activity and NSV chain typology categories.

SEEMS activity category	SII activities
Start‐up costs	
Materials development	Development of training materials, social and behaviour change communication materials, job aids
Training	One time only trainings at all levels for staff, frontline workers, individuals
Programme sensitization	Activities related to programme sensitization at all levels—national to community level
Programme installation	Hiring staff and frontline workers, start‐up meetings
Recruitment	Recruitment of staff and volunteers for SII activities
Planning	Planning activities included in start‐up phase
Recurrent costs	
Training (recurrent)	Recurrent or refresher trainings
Management	Project management meetings and activities
Monitoring	Establishing and using data systems for monitoring
Procurement	Procurement of chickens, seeds, agriculture tools and training materials on health/nutrition, agriculture, health, water/sanitation for distribution to programme participants, volunteers, households
Distribution of inputs	Distribution of chickens, seeds, agriculture tools and training materials to programme participants, volunteers, households, communities
Home visits: multisectoral interpersonal communication	Home visits conducted by frontline workers and staff for interpersonal communication, as part of SII social and behaviour change
Home visits for enhanced homestead food production	Home visits conducted by frontline workers (VMFs), and staff to provide technical assistance on agriculture/homestead food production
Health facility counselling and support	Health and nutrition support provided by the Health Post in Charge and Auxiliary Nurse Midwife at the health facility
Community events and mass media	Community‐level food demonstrations, agriculture field days, key life events, mass media campaigns, other community activities
Site supervision	National and subnational level supervision of implementation and service delivery
HMGs, adolescent groups, village model farm groups	Activities related to community groups (HMGs, village model farm groups, adolescent groups and so on)
Microcredit activities	Savings group activities
Marketing support	Activities related to strengthening market linkages
Integration and coordination	Recurrent review and planning meetings across implementing partners and the government, cross cutting activities including supporting GESI champions
Overhead/indirect costs	Office maintenance, office rent, phone, indirect personnel costs not directly related to above activities.
NSV chain typology category	
Increase demand	Activities designed to increase demand for nutritious foods and quality nutrition‐related services, primarily social and behaviour change interventions such as household visits, group meetings, key life events and mass media campaigns
Increase supply	Activities designed to enhance homestead food production and village model farm groups
Enabling environment	Activities enabling increased gender empowerment and government ownership and participation in all activities, focused on local nutrition governance and improved coordination between MSNP stakeholders.

Abbreviations: GESI, gender, equity and social inclusion; HMG, health mothers’ group; MSNP, Multisector Nutrition Plan; NSV, nutrition‐sensitive value chain; SII, *Suaahara* II; SEEMS‐Nutrition, Strengthening Economic Evaluations in Multisectoral Strategies for Nutrition; VMFs, Village Model Farmers.

We used mixed methods combining expenditures and micro‐costing to estimate the incremental total costs and unit costs of SII for the period July 2016 to March 2020 (3.7 years), which captured implementation costs before the onset of the coronavirus disease 2019 (COVID‐19) pandemic in Nepal. We estimated both financial costs (expenditures by HKI and subgrantees) and economic costs. We adopted a societal perspective, estimating costs for donated or shared resources, such as time use and out‐of‐pocket expenses incurred by frontline volunteers (e.g., FCHVs), government partners (e.g., health post staff) and participants (e.g., 1000‐day mothers), which were not already covered by SII project financial expenditures.

### 2.2 Study sample

The cost analysis was conducted in a sample of four districts enabling estimation of unit costs in four geographically diverse contexts within Nepal. The districts were purposefully selected for agroecological zone representation: the *Terai*/plains (Nawalparasi), hills (Bajhang, Dhading) and mountain (Sindhupalchok). Nawalparasi, Bajhang and Sindhupalchok were all SI and SII intervention areas; Dhading was only a SII intervention area and was included to more comprehensively understand start‐up costs. Within each district, we randomly sampled at least one urban and one rural municipality (Supporting Information S1: Appendix Table 3).

### 2.3 Data collection

Financial expenditure data were obtained from HKI accounting systems for all expenses incurred by HKI, including national and iNGO partners, and the local NGOs operating in the four sampled districts. We supplemented financial expenditure data with data collected through in‐depth interviews (IDIs) and focus group discussions (FGDs) with SII and local NGO programme staff on time and resource use. FGDs were used to estimate time and out‐of‐pocket expenses from government partners and frontline volunteers who supported SII implementation, but who were not paid by SII, and from programme participants.

IDIs and FGDs captured time spent on SII activities and additional out‐of‐pocket expenses incurred due to participation. A first round of qualitative data collection occurred in Dhading in September 2019, Sindhupalchok in January 2020, Nawalparasi in March‐April 2020, and Bajhang in July–August 2020. A second round of qualitative data collection occurred in all four districts in December 2021–February 2022 to increase the sample for government partners, frontline volunteers and programme participants. Interviews during the second phase were conducted over the phone due to COVID‐19 restrictions on movement, while FGDs were conducted in person.

#### 2.3.1 Outputs

Programme outputs were used to estimate unit costs for measuring reach of SII interventions: (1) the number of 1000‐day mothers reached; (2) the number of 1000‐day mothers combined with index children under 2 years of age reached (mother–child dyads); (3) the number of 1000‐day household members reached; and (4) the number of 1000‐day households reached by SII interventions. 1000‐day household members included grandparents, spouses, and non‐index children in the household. Households represented the number of unique households with a 1000‐day mother or child targeted by SII in each district. Project outputs were recorded as annual averages over the implementation period and provided the denominator for unit cost estimates, described below. Output data on participant and household reach were obtained from SII's monitoring systems and reports.

#### 2.3.2 Cost and time‐use estimates

Total incremental costs were defined as total incremental financial and economic costs, inclusive of all costs at national and subnational levels. Unit costs were defined as total incremental costs divided by programme outputs per 1000‐day mother, 1000‐day mother‐child dyad, total participant reached and household reached. We present the weighted average annual total incremental and unit costs.

We also present disaggregated cost data by cost type: economic or financial, by agroecological zone and by programme duration (‘new’ districts participating in SII compared to ‘continuing’ districts participating in SI and SII). We explored cost profiles to examine the share of total costs by input, activity and timing (startup or recurrent). Costs shares were explored by NSV typology and sector to describe resource allocation and distribution of costs necessary to implement SII. To understand community‐level contributions, we included monthly time estimates of participating frontline volunteers, government partners and 1000‐day mothers.

### 2.4 Data analysis

Using the SEEMS‐Nutrition standardized approach, we presented financial and economic costs separately to arrive at total and unit costs of the SII programme.

#### 2.4.1 Financial data

Financial data were analysed with a standardized SEEMS‐Nutrition expenditure analysis Microsoft Excel tool. We populated the tool with financial expense data for iNGO partners and local NGO partners in the four sampled districts. All line items were coded using SEEMS standardized input categories (Supporting Information S1: Appendix Table 2). Time allocation collected from interviews was used to allocate personnel costs to the SEEMS standardized activity, NSV typology and sector categories (Table 1). Capital costs were allocated based on similar allocation rules if costs were linked to specific personnel. For allocating nonlabor capital and recurrent input costs not linked to a specific activity or personnel role, we applied an average percentage based on how total personnel time was allocated across the SEEMS codes. We excluded expenditures related to research, international staff not involved in programme implementation and nonproject‐related activities. International staff involved in programme implementation such as country directors and management staff were included in the costs. We excluded international staff such as regional directors or global management staff who were not involved in programme implementation. All capital and start‐up costs were annualized using a 3% discount and useful years of capital cost were assumed to be 5 years. Financial data were recorded in Nepali rupees, then converted to 2020 USD using the relevant exchange rates for 2016–2019.

We analysed two tiers of financial costs: (1) National‐level financial costs, defined as SII expenditures incurred above the district level including HKI, international, and national partner financial costs and shared financial costs across the 42 districts and (2) subnational‐level financial costs, defined as all financial costs incurred by local NGOs operating at district, municipality, ward and community levels. We allocated a share of the national‐level costs to the subnational‐level financial costs based on the percentage of SII participants in each district to estimate total financial cost per district. Financial costs were presented on an annual basis by dividing the total costs for the programme period by 3.7 years.

#### 2.4.2 Economic costs

**Figure 1:**
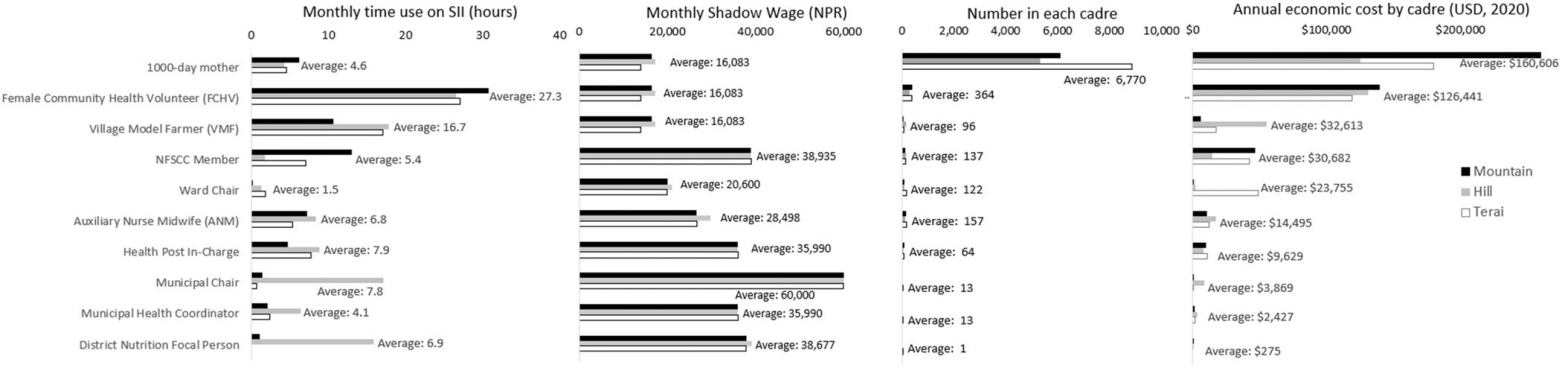
Monthly time use, shadow wages, number in each cadre and annual economic costs by ecological zone, Suaahara II.

We estimated economic costs for an average year, including average time spent on one‐time, monthly or recurrent activities for government and programme‐specific frontline workers, and participants engaged in SII activities. All estimates were standardized to average time (in hours) per month spent on SII activities for each cadre involved in the programme. Time estimates were multiplied by local wage rates for each cadre to value average monthly time use. We used district‐specific mean daily wage rates for municipal government officers, ward chairs, district nutrition focal persons and the unskilled labour wage rate for FCHV and participating mothers’ time (Figure 1). The value of each cadre and participant's time was combined with out‐of‐pocket expenses to estimate monthly economic costs by cadre and participant. These costs were scaled (multiplied by 12) to estimate annual economic costs. Cadre‐level average annual economic costs were multiplied by the number of individuals active in each district and summed to obtain district‐level average annual economic costs.

#### 2.4.3 Adjustments for frontline volunteers and participant economic costs

Several adjustments were necessary to obtain the incremental economic costs. First, as health mothers’ groups (HMGs) were active before SII started, we estimated that 10–15 min of typical 1 h HMG gatherings (~20%) was related to SII activities. This estimate was based on empirical observations during field visits and approved by the SII HKI implementation team. Hence, we adjusted FCHVs, CHVs and 1000‐day mothers’ reported time spent in HMG activities by 20%.

Second, we adjusted FCHV economic costs to reflect the reality that as uncompensated community facilitators, not all FCHVs were active volunteers. A monitoring survey conducted in 2017 by SII reported that 64.6% of FCHVs in the SII implementation area were active, so we only included economic costs for 64.6% of the total number of FCHVs in each district (Helen Keller International, 2019). Third, community nutrition volunteers (CNVs) received monthly stipends of ~US$133, which covered transportation, communication and time spent in SII activities. We did not include economic costs for CNVs, as their stipend was captured in financial expenditures. We also did not include time use costs for SII‐compensated personnel, as their salaries were captured as financial costs.

#### 2.4.4 Total and unit costs

**Figure 2:**
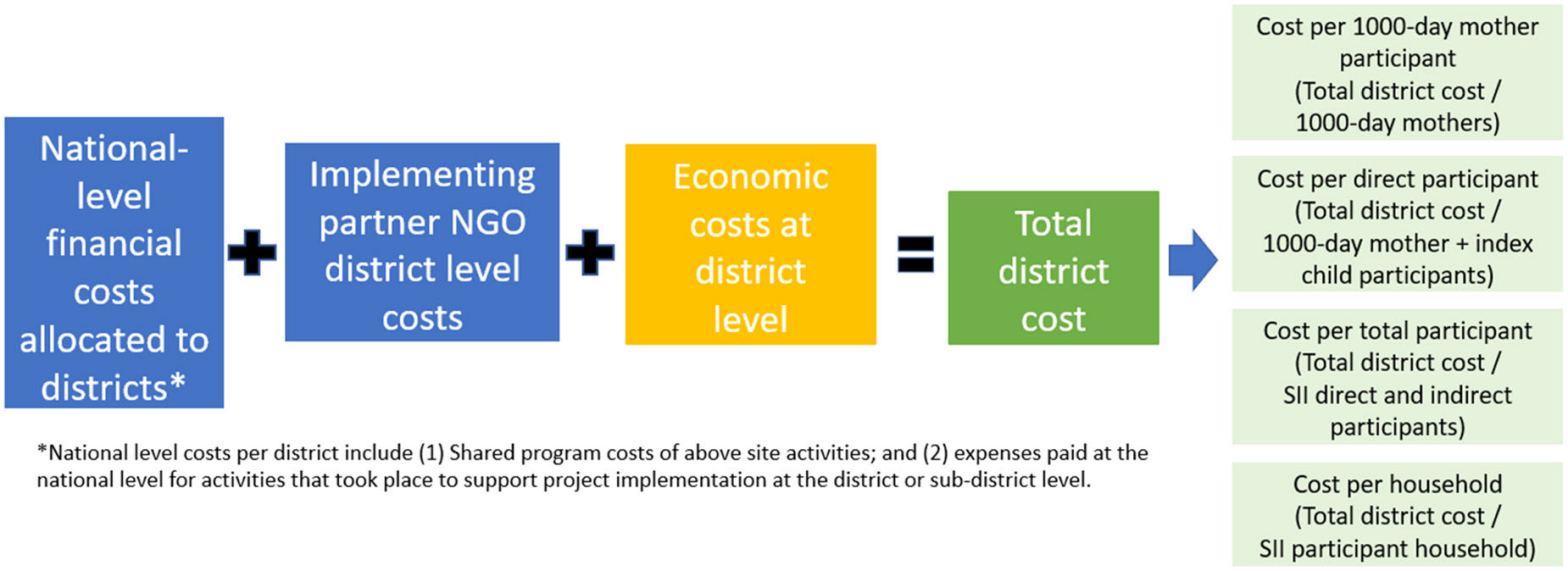
Aggregation of total costs and definition of unit costs, *Suaahara* II.

We estimated the annual total incremental cost for each district by summing annual financial and economic costs. We then estimated the weighted average total incremental cost using the percentage of the population living in each agroecological zone as follows: plains (46.7%), hills (43.1%), mountains (10.2%), based on National Population and Housing Census 2021 data (Government of Nepal, 2022). Weighted averages were used to reflect the representativeness of each district in the study sample compared to the total SII districts. As unit costs varied by agroecological zone, we estimated weighted average costs using the unit cost by zone and the share of SII populations living by zone over the total population engaged in SII. Annual incremental cost estimates are presented for the overall sample and by district, agroecological zone and length of programme involvement (Figure 2).

The cost per 1000‐day mother, per mother–child dyad, per total participant and per household reached was equal to the total incremental cost divided by the relevant programme outputs (1000‐day mother, mother–child dyad, total participant, household) for each district (Figure 2).

#### 2.4.5 Understanding government worker, frontline volunteer, and participant time

We analysed time use data for government workers, frontline volunteers, and participants to better understand drivers of economic costs. We used time allocation data on the frequency and duration of home visits, travel and other time spent to estimate average monthly time use in SII activities per district. We then calculated weighted average monthly time use estimates across the four sampled districts by actor type and SII activity. We compared government worker, frontline volunteer and participant time spent in activities by agroecological zone, because Nepal's diverse terrain presents different challenges in different areas, with effects on time use for programme implementation.

#### 2.4.6 Sensitivity analysis

Given the heterogeneity within and across districts in Nepal and the relatively small sample for estimating opportunity costs, we conducted a multivariate sensitivity analysis on volunteer and participant time and out‐of‐pocket costs to assess uncertainty in final cost estimates. We used a Monte Carlo probabilistic analysis to generate ranges for total and unit cost outcomes while varying underlying assumptions. We ran 5000 Monte Carlo simulations using Oracle Crystal Ball software version 11.1 (https://www.oracle.com/applications/crystalball) to draw from distributions specified for each parameter. We varied the following parameters using a gamma distribution, commonly used with costing data to non‐zero estimates (Dodd et al., 2006): (i) FCHV time in community events and household visits, (ii) FCHV roundtrip travel time to community events and household visits, (iii) FCHV out‐of‐pocket costs, (iv) 1000‐day mothers’ time in community events, (v) 1000‐day mothers’ roundtrip travel time to community events, and (vi) 1000‐day mothers’ out‐of‐pocket costs. For each parameter, we applied a gamma distribution parameterized with scale and shape estimates derived from the means and standard deviations from qualitative data (Supporting Information S1: Appendix Table 4). We varied:

1) Activity participation and travel time for FCHVs for community events and household visits given high variability in estimates across the sample.
2) Activity participation and travel time for 1000‐day mothers for community events because of uncertainty in estimates due to a small sample size and that community events were the overall largest share of economic costs.
3) Out‐of‐pocket costs for 1000‐day mothers and FCHVs because childcare, food, transportation, supplies and communication costs varied depending on agroecological zone.

We also conducted one‐way sensitivity analyses on time use for government workers to understand how increasing government involvement could affect total and unit costs. We increased time use for all five government cadres involved in SII (National Food Security Coordination Committee [NFSCC] member, Municipal Chair, Municipal Health Coordinator, Ward Chair, District Nutrition Focal Person) by 25% and by 200% to understand the impacts of varying government employee time on SII costs.

## 3 RESULTS

A total of 166 interviews and eight FGDs were conducted with 230 respondents (Supporting Information S1: Appendix Table 3). The average total incremental cost of SII was estimated to be US$908,948 per district per year for the period July 2016–March 2020 (Table 2a). Total incremental costs were highest in the plains and lowest in the hills at US$ $1,047,592 and US$ $754,207, respectively. On average, total financial cost per district was US$480,072. Economic costs contributed, on average, an additional US$428,876, comprising 47% of total costs (Table 2b). Economic costs were highest in the mountains, both in absolute terms (US$499,593) and in relative terms, having the highest cost share (54%) compared to other regions.

**Table 2a:** Average annual incremental costs for the *Suaahara* II project per district.

	Financial cost	Economic cost	Total cost	%
Input				
Personnel	$266,109	$303,012	$569,121	63%
Agriculture supplies	$6289	$0	$6289	1%
Equipment	$6091	$0	$6091	1%
Contracted services	$34,843	$0	$34,843	4%
Transportation	$5172	$0	$5172	1%
Travel/per diem	$32,898	$38,047	$70,946	8%
Other supplies	$32,065	$70,881	$102,946	11%
Overhead	$69,466	$0	$69,466	8%
Mixed inputs	$27,139	$16,935	$44,074	5%
Total	$480,072	$428,876	$908,948	100%
Stage				
Start‐up	$30,244	$16,697	$46,941	5%
Recurrent	$449,827	$412,179	$862,006	95%
Total	$480,072	$428,876	$908,948	100%
Activity				
Planning	$21,786	$2610	$24,396	3%
Programme Installation	$1,486	$2610	$4097	0.5%
Programme sensitization	$3765	$0	$3765	0.4%
Recruitment	$1470	$0	$1470	0.2%
Training	$57,576	$11,476	$69,052	8%
Materials development	$18,692	$0	$18,692	2%
Management	$33,852	$909	$34,761	4%
Monitoring	$37,590	$583	$38,173	4%
Procurement	$2835	$0	$2835	0.3%
Distribution of inputs	$8020	$0	$8020	1%
Site supervision	$28,019	$1589	$29,608	3%
Home visits: multisectoral interpersonal communication	$21,420	$134,667	$156,086	17%
Home visits for enhanced homestead food production	$7284	$8096	$15,381	2%
Health facility counselling and support	$3742	$0	$3742	0.4%
Community events and mass media	$45,137	$219,037	$264,174	29%
Health mothers’ groups, adolescent groups, village model farm groups	$13,216	$46,950	$60,166	7%
Microcredit activities	$1526	$0	$1526	0.2%
Marketing support	$1502	$0	$1502	0.2%
Integration and coordination	$24,521	$349	$24,870	3%
Overhead/Indirect costs	$146,633	$0	$146,633	16%
Total	$480,072	$428,876	$908,948	100%
Sector				
Nutrition	$100,740	$171,557	$272,297	30%
Health	$92,110	$170,297	$262,407	29%
Water, sanitation, and hygiene	$81,506	$145	$81,651	9%
Agriculture, poultry, livestock	$26,476	$32,867	$59,343	7%
Market sector	$17,898	$0	$17,898	2%
Inter‐sector	$161,342	$54,009	$215,351	24%
Total	$480,072	$428,876	$908,948	100%

**Table 2b:** Weighted total costs for the SII programme per district per year, USD 2020.

	Financial cost per district (US$)	Economic costs per district (US$)	Total cost per district (US$)	Financial cost (%)	Economic cost (%)
Overall	$480,072	$428,876	$908,948	52.8%	47.2%
By agroecological zone					
Mountain	$428,221	$499,593	$927,814	46.2%	53.8%
Hill	$378,767	$375,440	$754,207	50.2%	49.8%
Terai	$584,845	$462,748	$1,047,592	55.8%	44.2%
By length of SII involvement					
New district	$395,899	$335,483	$731,383	54.1%	45.9%
Continuing districts	$472,688	$446,096	$918,784	51.4%	48.6%

Abbreviation: SII, *Suaahara* II.

When considering the number of participants and households reached each year, the average unit costs were US$132 per 1000‐day participating mother, US$76 per mother–child dyad, US$10 per total participant and US$140 per household reached (per year). The highest unit costs were in the mountain districts ($152) and lowest unit costs in the plains (US$118) per 1000‐day mother (Table 3b). The average annual cost per household was also highest in the mountains (US$159) and lowest in the plains (US$125).

**Table 3:** Incremental annual unit costs for the SII project, USD 2020.

A. Number of annual beneficiaries per district					
	Target participations			Total participants (direct and indirect^a^)	Households
	1000 day mothers	Index child (child < 2)	Total direct (mother + index child)		
Overall	7063	5049	12,112	99,419	6639
Agro‐ecological zone					
Terai	8874	6131	15,005	132,472	8359
Hill	5326	3919	9245	67,623	4963
Mountain	6111	4870	10,981	82,380	5848
Length of programme participation					
Continuing district	7078	5097	12,175	95,218	6599
New district	5292	3807	9099	77,371	5056

**B. Annual unit costs by district and participant type**					
	**1000‐day mother**	**Child 0–23.9 months**	**1000‐day mother + child 0–23.9 months**	**Total participants (direct and indirect** a **)**	**Households**
Overall	$131.64	$182.17	$76.38	$9.77	$140.26
Agro‐ecological zone					
*Terai*	$118.05	$170.88	$69.82	$7.91	$125.33
Hill	$141.59	$192.45	$81.56	$11.44	$152.10
Mountain	$151.83	$190.53	$84.50	$11.26	$158.65
Length of programme participation					
Continuing districts	$133.10	$182.31	$76.89	$10.63	$143.47
New district	$138.20	$192.13	$80.38	$9.45	$144.66

Abbreviation: SII, *Suaahara* II.

^a^ Household members reached through any SII activity including mass media.

### 3.1 Cost drivers

**Figure 3:**
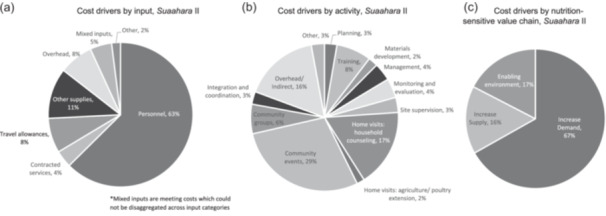
(a) Cost drivers by input, *Suaahara* II. (b) Cost drivers by activity, *Suaahara* II. (c) Cost drivers by nutrition‐sensitive value chain, *Suaahara* II.

Recurrent activities drove most of the costs, with only 5% of costs allocated to start‐up activities (Table 2a). The main cost drivers by input were personnel (including volunteer) costs (63%), followed by supplies (11%), travel (8%), overhead (8%), mixed inputs (5%) and contracted services (4%) (Figure 3a). Mixed inputs encompassed meeting and training costs in which costs could not be disaggregated across input categories. Less than 5% of costs comprised of expenses related to agriculture supplies, equipment and transportation. For costs by activity, community events accounted for 29% of total annual costs, followed by home visits for counselling (17%), overhead (16%) and training (8%) (Figure 3b).

According to the NSV chain typology, the majority of SII programme activities focused on increasing demand for nutritious foods (67%), 17% of costs were incurred to strengthen the enabling environment for healthy nutrition and 16% to increasing the supply of nutritious foods (Figure 3c). Mapping costs by sector, nutrition and health sector‐related activities accounted for ~60% of costs followed by inter‐sector costs (24%). Inter‐sector costs included coordination, disaster‐risk reduction activities, governance, and gender, equity and social inclusion costs. Notably, agriculture and market sector activities comprised <10% of costs (Table 2a).

### 3.2 Economic costs

#### 3.2.1 Time use in SII activities

FCHVs had the highest monthly time use among all implementing cadres and programme participants (Supporting Information S1: Appendix Figure 3). FCHVs spent an average of 27 h per month on SII, ranging from 31 h in the mountains to 27 h in the plains (Supporting Information S1: Appendix Figure 3). This was equivalent to spending an average of 90% (range: 68%–135%) of their work time on SII‐related activities based on a 5–10 h work week (Singh et al., 2015). FCHVs spent 48% of their SII time organizing community events, 40% on household visits and 12% facilitating HMG meetings. Travel time consisted of 22% of overall FCHV time on SII implementation, ranging from 16% in the plains to 28% in the hills. VMFs had the second highest time use and spent an average of 17 h per month in agriculture, specifically enhanced homestead food production, and support to households (12 h on activities and 5 h on travel) (Supporting Information S1: Appendix Figure 3). Among VMF time spent on SII, home visits were the most time consuming, taking an average of 9 h per month.

Among government workers, municipal chairpersons spent 8 h and district‐level nutrition focal persons spent 7 h per month supporting SII activities (Supporting Information S1: Appendix Figure 3). This was equivalent to 17%–20% of a typical week based on a 40 h work week. Government workers based in hill and mountain areas spent more time in SII per month compared to those based in the plains. Across all agroecological zones, government workers spent the majority of their time supporting implementation of SII at the community level.

In terms of participants, 1000‐day mothers spent 5 h per month on SII activities, spending the majority of their time on community events and in HMG and VMF meetings (Supporting Information S1: Appendix Figure 3). This time use estimate translated to about 4% of a typical work week, based on an assumed 6 h day. Travel comprised 12% of total participating mothers’ time spent on SII activities.

#### 3.2.2 Total economic costs by actor type

Total economic costs were highest in mountain districts and lowest in hill districts (Table 2b). Here, 1000‐day mothers had the highest share of total economic costs (37%) followed by FCHVs (30%), VMFs (8%) and NFSCC members (7%) who support nutrition governance and coordination (Supporting Information S1: Appendix Figure 4).

Although average wage rates for volunteers and women participants were lower than paid programme staff or government stakeholders, FCHV and 1000‐day mothers contributed the highest shares to economic costs (Figure 1). VMFs contributed the third highest share of economic costs with the second highest time use among all cadres. NFSCC members bore the fourth highest share of economic costs with an average of 137 members participating in SII activities per district, coupled with the second highest shadow wage.

### 3.3 Sensitivity analyses

**Figure 4:**
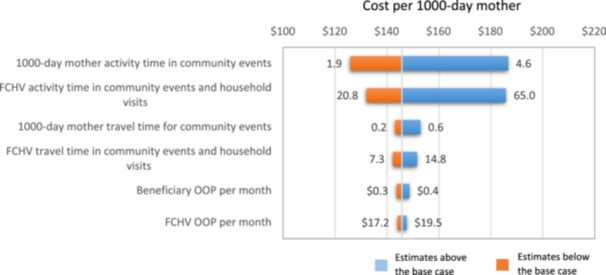
Tornado diagram for sensitivity analysis of unit incremental cost (cost per 1000‐day mother, *Suaahara* II).

When we varied parameters in sensitivity analyses, total incremental costs ranged from a lower bound of US$1,149,466 to a higher bound of US$4,476,848 and the unit cost per 1000‐day mother ranged from US$171 to US$698 per year. The tornado plot (Figure 4) depicts variables with their corresponding magnitude of influence on incremental cost per participant. Uncertainty in time spent in community events by 1000‐day mothers (range of 1.9–4.6 h per month) had the largest impact on the range of costs, followed by FCHV activity time in community events and household visits.

To explore varying government worker involvement in SII, one way sensitivity analysis showed that the cost per 1000‐day mother increased to $135.36, a 1.8% increase from the base case and total cost increased by 2% ($925,968) if NFSCC member time spent on SII was doubled in each sampled district (Supporting Information S1: Appendix Table 5). Increasing other government workers’ time by double changed total and unit costs by less than 1%.

## 4 DISCUSSION

This study captured the total incremental costs of SII, a USAID‐funded, at scale multisectoral nutrition programme in support of Nepal's MSNP. Implemented by both international and local NGOs, SII invested in interventions to increase awareness and improve practices related to the production and consumption of nutritious foods, improve access and quality of health and nutrition services, and strengthen nutrition governance at national and subnational levels, to ultimately reduce maternal and child undernutrition. Implemented in 42 of Nepal's 77 districts (389 municipalities), SII reached over two million 1000‐day mothers and children, and an additional nine million household members using a family‐centred approach that relied on community SBCC interventions to create a supportive and enabling environment to improve maternal and child nutrition outcomes. Consistently reaching 1000‐day mothers and their families required an intervention that was complex in scope, spanning multiple sectors with multiple points of contact, implemented at multiple administrative levels (national and subnational) and reliant on management and technical support from HKI, six international partners and nearly 40 local district community‐based organizations.

We applied the SEEMS‐Nutrition standardized costing approach to facilitate comparisons with other multisectoral nutrition programmes and to capture the full range of resources required for implementation. The average annual cost of reaching 1000‐day mothers was $132 per woman and reduced to $76 when considering their children under 2 years of age. These unit costs of SII are similar to other multisectoral nutrition interventions in Malawi and Bangladesh that used the SEEMS approach, with costs ranging from US$112 to $160 per participant. (Gelli et al., 2022; Thai et al., 2023) Although these programmes vary in target population and programme components, they all share a strong focus in SBCC activities to change knowledge, attitude and practices for producing and consuming nutritious foods. When considering the larger reach of SII to the broader community, beyond mother–child dyads, the unit cost of SII as a community wide programme declines significantly. The cost of reaching both total household members (~11 million, or typically around 80% of the total district population) was $10, with the lowest unit costs in districts based in the plains at $8 per individual. While SII's intervention components and unit costs per participant were comparable to other multisectoral nutrition interventions (De Brauw et al., 2018; Gelli et al., 2022; Levin, Self, et al., 2019; Thai et al., 2023), its scale was much larger than similar cost studies published to date, which are often derived from pilot or demonstration projects or randomized controlled trials. Assessing the costs of a scaled programme illustrates the advantage of economies of scale when integrating programmes into existing health and governance infrastructure.

Considering costs by agroecological zone or other meaningful programme characteristics provides insights for programmes implemented nationally or outside of Nepal. In a scaled‐up programme that spanned several investment phases, bringing new districts from one phase to the next may have some benefits in keeping costs low. Although the average unit costs for new districts that joined later in SII were higher than established districts from SI, the difference was marginal. New districts introduced in SII benefited from a well‐honed programme, training workshops, materials, monitoring systems and existing community‐based processes. The increase in overall costs reflected higher opportunity costs (resource use) by new participants. When exploring resource allocation across nutrition sensitive intervention typology, SII allocated over 65% of all resources to improve the demand for nutritious foods, slightly higher than programmes in Malawi (53%) and Bangladesh (50%) (Margolies et al., 2021; Thai et al., 2023).

Capturing the economic or opportunity costs of frontline volunteers and participants is critical for estimating total resources required to implement multisectoral interventions supported by local governance and communities themselves. Our analysis showed that economic costs comprise on average 47% of total costs and up to 56% in mountainous zones, slightly higher than other cost studies (Margolies et al., 2021; Thai et al., 2023). Total financial costs were highest in the plains, with higher population density and greater programme reach, and lowest in the remote mountain region, with lower density and fewer participants reached. Yet, when including the opportunity costs of time, including travel, total economic costs were highest in the mountains. Unit costs were also highest in the mountains, reflecting greater travel time in mountain areas due to household dispersion and access constraints such as distance and poor roads. In the plains region, the share of economic cost was lowest, as were costs per participant. Estimating both financial and economic costs demonstrates that some districts may need extra resources to facilitate greater participation. Additionally, including opportunity costs may help to identify efficient or lower cost delivery strategies that reduce time burden, reduce costs and improve reach.

Complex multisectoral programmes often require significant resources to ensure coordination and sufficient technical support is provided to implementers at the district‐level or below. SII not only coordinated across sectors, but also across implementation levels from national to subnational to identify and strengthen collaborations. Using a multisectoral and multilevel approach facilitates synergies across stakeholders but requires ample investment in personnel costs, integration and coordination costs. High personnel costs are common in these programmes, as large numbers of staff are involved in implementation across sectors (Levin, Masters, et al., 2019; Margolies et al., 2021; Thai et al., 2023). As noted, SII's inclusion of a wide range of activities across sectors required engagement of diverse organizations with experience and expertise spanning multiple sectors and levels. Technical assistance, management, coordination and oversight of this large‐scale programme accounted for 53% of total costs. As working closely with communities has implications for time use and management, we explored how SII affected frontline volunteers, government workers and participants' time. First, SII activities were designed to be integrated into existing rural and urban community health extension networks. The programme leveraged an existing FCHV network, a key component of Nepal's health system, equipping them with greater tools to reach households including directed trainings (Ministry of Health–Nepal, 2010). Therefore, understanding the time invested by FCHVs in SII was important to consider for Nepal's expansion of their national MSNP. FCHVs spent 28 h per month on SII activities, falling within the range from other studies on community health volunteer time use. Unpaid Community Health Workers (CHWs) in Uganda had a monthly workload of 19.3 h, while part‐time paid CHWs in rural India spent 25.3 h per week on health and nutrition activities (Garg et al., 2022; Kasteng et al., 2016). In Nepal, FCHVs typically volunteer 5–10 h per week, suggesting that SII activities likely complemented existing FCHV duties including immunization and vitamin A supplementation (Government of Nepal Ministry of Health and Population, 2019; Ministry of Health–Nepal, 2010; Singh et al., 2015). Using this range of work hours, some FCHVs involved in SII may have increased their work hours to accommodate for a higher workload (7 more hours per month, a 35% increase). Thoughtful partnerships between implementers and FCHVs are vital to avoid overburdening community volunteers.

Second, we explored the impact of SII on government worker time. Since government time spent on SII was relatively low, we used sensitivity analysis to double their total SII‐related time commitment, revealing that this change in time use did not affect overall costs substantially. Although there was a limited number of government workers involved in direct implementation of SII, especially at the municipality level and above, transitioning to government led MSNP implementation in these 42 districts would require more extensive involvement of government workers to coordinate, manage and ensure collaboration across sectors and implementation levels. Strengthening coordination bodies such as local NFSCCs may be a key approach to accelerate nutrition governance and action.

Third, we explored 1000‐day mothers time in SII activities. On average, 1000‐day mother participants spent 4.6 h per month, or <3% of a typical working month, in programme activities. This estimate is lower than other time use studies of nutrition‐sensitive programmes involving women (van den Bold et al., 2021). Understanding costs associated with measured changes in infant and young child feeding, such as improved dietary practices, suggests that engaging mothers frequently through regular interaction, not necessarily requiring extensive time, may be an effective approach to provide support in the first 1000 days (Frongillo et al., 2024).

This study had several limitations. Cost data were collected from four of 42 SII implementation districts due to budget and logistical constraints. To address representativeness, we sampled districts across agroecological zones and included both new and previously supported districts to maximize variation. We did not scale cost estimates to the full 42 districts as we did not want to introduce bias due to our sampling strategy. Qualitative data were collected in different seasons to capture average time use across seasons, but limited interpretations can be made for opportunity costs within a single season. Increases in women's opportunity cost of time as a result of participation in nutrition‐sensitive programmes during peak agriculture season has been shown to negatively impacted women's dietary intake, but this study did not capture resource use linked to seasonal agricultural activities (Vemireddy & Pingali, 2021). In addition, the mode of data collection for interviews changed due to COVID‐related restrictions on travel from in person to over the phone. To ensure data comparability, we asked about typical time spent on activities before COVID restrictions during phone interviews, but recall bias may have influenced results. Lastly, as this analysis estimates the full costs of SII incremental to services and materials developed as part of SI (2011–2015), our analysis may underestimate the full start‐up costs that were carried over to SII, such as awareness raising, establishing local offices, and materials development. As these costs are considered a type of fixed costs, the longer the project extends, the magnitude of the unit costs is likely to diminish over time and with scale. While SII may underestimate some of the start‐up costs, which typically range from 5% to 23% in similar multisectoral programmes, our estimates capture 5% of start‐up cost and are robust for representing the ongoing full implementation costs of a programme at scale (Margolies et al., 2021; Thai et al., 2023).

Further analysis comparing SII costs to the government's cost of implementing the MNSP could help to assess affordability of an enhanced government owned and implemented programme like SII. Similarly, a future analysis comparing the costs to measured impact outcomes will provide additional evidence on return on investment for SII and can inform donor and government planning and decision making.

This study provides evidence on the costs of SII, a scaled‐up multisectoral nutrition programme, including insights into opportunity costs for health volunteers and programme participants. Costs of these programmes can be substantial and vary by agroecological zone and reach, especially when considering opportunity costs. Estimating both financial and economic costs can better inform programme design, budgeting, and resource allocation for scaled up programmes, taking into consideration variations in population density, geographic location and programme participation. Initiatives such as SEEMS‐Nutrition are contributing to expanding the evidence base around the costs of multisectoral nutrition strategies with standardized guidance to conduct these studies.

## AUTHOR CONTRIBUTIONS

Carol Levin, Christopher G. Kemp and Kenda Cunningham designed the study. Carol Levin and Christopher G. Kemp developed the cost study protocol and methods. Christopher G. Kemp, K. C. Sagun, Uttam Paudel and Pushpa Acharya led or participated in data collection efforts. Esther M. Choo, Jolene Wun and Christopher G. Kemp conducted data analysis. K. C. Sagun, Kenda Cunningham and Pooja Pandey contributed to data interpretation. Esther M. Choo and Carol Levin wrote the original draft. All authors reviewed, edited and approved the final version.

## CONFLICT OF INTEREST STATEMENT

The authors declare no conflict of interest. K.C. Sagun, Kenda Cunningham and Pooja Pandey Rana were members of the *Suaahara* II programme team that designed and implemented the interventions discussed in this article. They reviewed the manuscript and provided interpretation of the results, but final decisions for content were made by the primary authors from the research and analysis team (Esther M. Choo, Christopher G. Kemp, Carol Levin). The US Agency for International Development participated in the design of the study and provided feedback throughout the study but did not participate in the data collection or analysis.

## Supporting information

Supporting Information

## Data Availability

The data that support the findings of this study are available from the corresponding author upon reasonable request.
