# Supplementary material for: The costs of Suaahara II, a complex scaled‐up multisectoral nutrition programme in Nepal
Source: Matern Child Nutr. 2024 May 5;22(1):e13658. doi: 10.1111/mcn.13658 (PMC12647970; doi:10.1111/mcn.13658)
Supplement: Supplementary file 1 — Supporting Information [file MCN-22-e13658-s001.docx]

**Supplementary Appendix**

Figure 1: *Suaahara* II Theory of Change


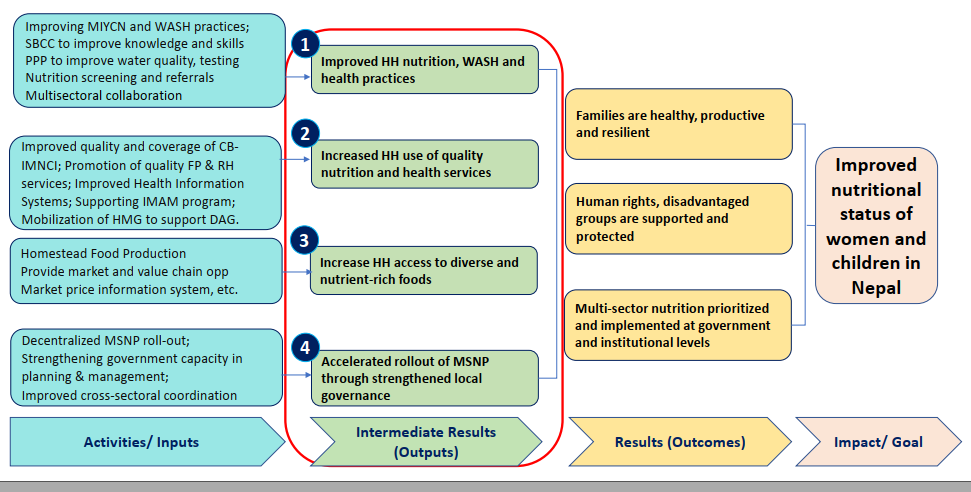


Figure 2: *Suaahara* II CORE and CORE+ components

CORE package applies to all the *Suaahara* II intervention wards, while CORE+ applies to 45% of the intervention wards.

Table 1. Cadres and participants involved in the *Suaahara* II (SII) program activities.

| **Category** | **SII role** | **Description of involvement in SII** |
| --- | --- | --- |
| Program participants | 1000-day mothers | The SII program focused on the 1000-day period with pregnant women and mothers of children < 2 as target program participants. 1000-day mothers participated in behavior change activities at their homes and during community events. Activities included learning about improved practices for nutrition, health, WASH, and homestead food production aimed at improving the well-being of both the mother and baby. |
|  | Household members | Indirect program participants include grandparents, husband/partners of 1000-day mothers, and other children in the household. |
| Frontline worker | FCHV | FCHVs facilitated health mothers’ groups (HMGs), conducted home visits, and reporting. SII supported FCHVs to organize community events, such as food demonstrations and Enhanced Homestead Food Production (EHFP) demonstrations. |
|  | CNV | Community Nutrition Volunteers (CNVs) identified mothers in the 1000-day period, supported frontline workers, such as FCHVs and healthcare professionals at Primary Health Centers (PHC), as well as at outreach and immunization clinics. CNVs also support Village Model Farmers (VMFs) and Household Food Production (HFP) groups. |
|  | Field supervisor | Field supervisors (FS) were SII field staff responsible for implementing program activities. FS coordinated with government officials and other stakeholders at the community level. They planned and executed *Suaahara* II activities, overseeing the work of Community Nutrition Volunteers (CNVs), monitoring the progress, providing support FCHVs, outreach clinics, and Community-Based Organizations (CBOs), ensuring timely reporting of activity progress. |
|  | Health post in charge | The SII program staff trained healthcare workers at local health facilities to deliver better maternal and child health, as well as nutrition services. Throughout the program, healthcare workers (Health post in-charge, ANM) actively participate in different on-site coaching sessions. |
|  | Auxiliary Nurse Midwife (ANM) |  |
|  | VMF | Village Model Farmers are progressive women leaders, 1000-day mothers, and Female Community Health Volunteers. VMFs receive diverse training focused on group management, social mobilization, larger-scale vegetable and poultry farming, agricultural marketing, and climate-smart agricultural technology demonstration. VMFs play a crucial role as catalysts for improving the production of nutritious food within their communities. They receive ongoing technical support and contribute by providing vegetable seeds or chicks from their own production. Additionally, VMFs collaborate with local municipal agriculture and livestock units to leverage government resources. |
| Government workers | Municipal health coordinator | SII supported the Government of Nepal (GoN) at three levels of government in designing, implementing, and rolling out the GoN Multisector Nutrition Plan (MSNP). SII fostered local ownership of the program by engaging various government officials in planning, coordination, implementation, monitoring, technical visits, and program activity reviews. Government officials received training in nutrition governance and budgeting initiatives. Government workers also actively participate in SII community events, including fairs, exhibitions, promotion campaigns, and various celebrations. |
|  | Municipal chair |  |
|  | Ward chairperson |  |
|  | District Nutrition Focal point |  |
|  | NFSCC members |  |

| Table 2. SII inputs mapped to SEEMS-Nutrition input categories | |
| --- | --- |
| **SEEMS Input category** | **SII Inputs** |
| Personnel | *Suaahara* II staff paid time, paid and volunteer frontline workers, participant time |
| Equipment | Capital goods including laptops and vehicles |
| Contracted services | Contracts for venues and rental to support training. Rental and support costs to training |
| Transportation | Fuel, maintenance |
| Travel/per diem | Travel allowances, per diems |
| Other supplies | Office supplies, refreshments |
| Overhead | Facilities and office rent, communication costs |

Table 3: Qualitative sample by district for *Suaahara* II cost study

| **Category** | **Cadre** | **Dhading** | | **Sindhupalchok** | | **Nawalparasi** | | **Bajhang** | | **Total** |
| --- | --- | --- | --- | --- | --- | --- | --- | --- | --- | --- |
|  |  | **Urban** | **Rural** | **Urban** | **Rural** | **Urban** | **Rural** | **Urban** | **Rural** |  |
| **Beneficiaries** | **1000-day mother participant** | 6 | 16 | 5 | 5 | 6 | 7 | 10 | 9 | 64 |
| **Frontline** | **Female Community Health Volunteer** | 9 | 9 | 2 | 2 | 2 | 2 | 2 | 2 | 30 |
|  | **Community Nutrition Volunteer** | 6 | | 2 | 2 | 2 | 1 | 2 | 2 | 17 |
|  | **Field Supervisor** | 6 | | 2 | 1 | 1 | 1 | 2 | 2 | 15 |
|  | **Health post in charge** | 1 | 2 | 2 | 0 | 2 | 2 | 1 | 1 | 11 |
|  | **Auxiliary Nurse Midwife** | 1 | 1 | 0 | 1 | 2 | 2 | 2 | 2 | 11 |
|  | **Village Model Farmer** | n/a | 1 | 2 | 1 | 1 | 1 | 1 | 1 | 8 |
| **Government** | **Health coordinator** | 1 | 2 | 2 | 1 | 2 | 2 | 1 | 1 | 12 |
|  | **Municipal chairperson** | 1 | 2 | 1 | 0 | 1 | 1 | 0 | 0 | 6 |
|  | **Ward chairperson** | 0 | 2 | 2 | 1 | 1 | 1 | 1 | 1 | 9 |
|  | **District nutrition focal person** | 1 | | 1 | |  | |  | | 2 |
|  | **NFSCC member** | 0 | 2 | 2 | 1 | 3 | 3 | 1 | 1 | 13 |
| **Staff** | **Local NGO finance officer** | 1 | | 1 | | 1 | | 1 | | 4 |
|  | **Local NGO field coordinator** | 1 | | 1 | | 1 | | 1 | | 4 |
|  | **Local NGO Executive Director** |  | | 1 | | 1 | | 1 | | 3 |
|  | **SII finance officer** | 1 | | 1 | | 1 | | 1 | | 4 |
|  | **SII HFP officer** | 1 | |  | | 1 | | 1 | | 3 |
|  | **SII MNCH/GESI officer** | 1 | | 1 | | 1 | | 0 | | 3 |
|  | **SII NSBCC officer** | 1 | | 1 | | 1 | | 1 | | 4 |
|  | **SII program coordinator** | 1 | | 1 | | 1 | | 1 | | 4 |
|  | **SII WASH officer** | 1 | | 0 | | 1 | | 1 | | 3 |
| **Total respondents** | | | | | | | | | | 230 |

Abbreviations. SII: *Suaahara* II program; HFP: Homestead Food Production; MNCH: Maternal, Newborn, and Child Health; GESI: Gender and social inclusion; NSBCC: Nutrition social behavior change communication; WASH: water, sanitation, and hygiene

Figure 3: Monthly time use on *Suaahara* II by cadre (hours)

Abbreviations. FCHV: female community health volunteer, VMF: Village Model Farmer, ANM: Auxiliary Nurse Midwife,

NFSCC: Nutrition and Food Security Coordination Committee

Figure 4: Economic cost by cadre and participant, *Suaahara* II

Abbreviations. NFSCC: Nutrition and Food Security Coordination Committee

Table 4: Multivariate sensitivity analysis parameters and results for *Suaahara* II cost study

| **Parameters** | **Mean** | **Standard deviation** | **PSA**  **Distribution** | |
| --- | --- | --- | --- | --- |
| **1000-day mother** | | | | |
| Activity time in community events per month (hours) | 1.53 | 1.18 | Gamma | |
| Travel time for community events per month (hours) | 0.17 | 0.17 | Gamma | |
| Out of pocket costs per month (US$)** | $0.2 | 0.07 | Gamma | |
|  |  |  |  | |
| **Female Community Health Volunteer** | | | | |
| Non-HMG activity time per month (hours) | 19.09 | 20.29 | | Gamma |
| Non-HMG travel time per month (hours) | 5.27 | 3.06 | | Gamma |
| Out of pocket costs per month (US$)** | $10.45 | 1.18 | | Gamma |

*Abbreviations. HMG: Health Mothers Group, PSA: Probabilistic sensitivity analysis, FGD: Focus group discussion

** Out of pocket costs per month for 1000-day mothers was 0.14% of the local monthly wage. Monthly out of pocket costs for Female Community Health Workers was 7.14% of the local monthly wage.

Table 5: One way sensitivity analysis varying time use for government workers in *Suaahara* II (USD 2020)

|  | **Total cost** | **Cost per 1000-day mother** | **Cost per direct participant (mothers + child)** | **Cost per direct + indirect participants** | **Cost per household** |
| --- | --- | --- | --- | --- | --- |
| **Base case (mean)** | $908,491 | $131.64 | $76.38 | $9.77 | $140.26 |
|  |  |  |  |  |  |
| **NFSCC member** |  |  |  |  |  |
| Increase time use by 25% | $912,867 | $133.57 | $77.50 | $9.90 | $142.30 |
| Double time use | $925,968 | $135.36 | $78.54 | $10.03 | $144.20 |
|  |  |  |  |  |  |
| **Ward chair** |  |  |  |  |  |
| Increase time use by 25% | $909,123 | $133.06 | $77.21 | $9.87 | $141.76 |
| Double time use | $911,018 | $133.32 | $77.36 | $9.88 | $142.03 |
|  |  |  |  |  |  |
| **District Nutrition Focal Person** | | | | | |
| Increase time use by 25% | $908,512 | $132.98 | $77.16 | $9.86 | $141.67 |
| Double time use | $908,574 | $132.99 | $77.17 | $9.86 | $141.68 |
|  |  |  |  |  |  |
| **Municipal chair** |  |  |  |  |  |
| Increase time use by 25% | $908,995 | $133.07 | $77.21 | $9.87 | $141.76 |
| Double time use | $910,504 | $133.34 | $77.37 | $9.88 | $142.05 |
|  |  |  |  |  |  |
| **Municipal Health Coordinator** | | | | | |
| Increase time use by 25% | $908,817 | $133.03 | $77.19 | $9.86 | $141.72 |
| Double time use | $909,793 | $133.19 | $77.28 | $9.87 | $141.89 |

Abbreviations. NFSCC: Nutrition and Food Security Coordination Committee
